# Public Attitudes Towards Vaccine Passports in Alberta During the “Pandemic of the Unvaccinated”: A Qualitative Analysis of Reddit Posts

**DOI:** 10.3389/ijph.2023.1606514

**Published:** 2023-12-22

**Authors:** Bobbi Rotolo, Gaya Bin Noon, Helen Hong Chen, Zahid Ahmad Butt

**Affiliations:** School of Public Health Sciences, University of Waterloo, Waterloo, ON, Canada

**Keywords:** COVID-19, vaccine mandates, vaccine passports, public attitudes, Alberta, Canada, Reddit

## Abstract

**Objective:** The goal of this study is to understand the attitudes and beliefs towards mandatory vaccination policies in Alberta, Canada in September 2021, during the fourth wave of COVID-19.

**Methods:** 9400 posts between 1st September and 30th September 2021 were collected from the subreddit r/Alberta with Pushshift.io. Posts and comments were manually screened to determine their relevance to research objectives, and then coded using inductive coding and iterative qualitative analysis methods.

**Results:** Inductive coding methods yielded five key themes: 1) opinions related to autonomy and consent, 2) concerns about COVID-19 vaccine passport enforcement, 3) concerns about government, 4) concerns about the logistics of passports, and 5) opinions relating to the necessity of passports to prevent lockdowns.

**Conclusion:** Overall, the data presented favorable opinions towards an Albertan vaccine passport within r/Alberta. Anti-vaccine and anti-mandate opinions were often less extreme than those present in the literature, although this may be due to r/Alberta subreddit moderators removing those more extreme comments. Most reservations were due to issues of bodily autonomy, though concerns about the government and logistics also played a meaningful role.

## Introduction

In September of 2021, Alberta was in the midst of what their then-Health Minister Tyler Shandro referred to as a “pandemic of the unvaccinated”: the number of COVID-19 cases surged across the province, with unvaccinated groups being the hardest hit [1, 2]. During this time, Alberta had nearly four times the national average of cases and Albertans were dying of COVID-19 at nearly three times the rate of any other province, with the exception of Saskatchewan [2].

While it may be partially due to the fact that, by the end of September 2021, Alberta and Saskatchewan were the only two provinces with a cumulative percent below 70% of people with at least one dose of a COVID-19 vaccine (the national percentage being 75% at the time), political factors may have also played a big part in these high rates [2–4]. That previous June, the Alberta government, led by then-Premier Jason Kenney of the United Conservative Party (UCP), implemented a staged “Open for Summer” reopening plan based on vaccination thresholds [4, 5]. With the release of this plan, Premier Kenney said that once Alberta was “open” it would be “open for good,” a claim which he later admitted was premature and apologized for [6]. This reopening was followed by a severe fourth wave during which Alberta suffered more cases, hospitalizations, critical care, and deaths, and the provincial healthcare system was overwhelmed [7].

Around the same time, other Canadian provinces were implementing proof-of-vaccination certificates that individuals eligible for vaccination would be required to present before entering certain public settings [8–11]. Premier Kenney strongly opposed implementing a similar program, instead initially offering a $100 incentive for vaccination [2, 4]. He reversed this decision among rising case counts in mid-September, creating a passport system under the name “restrictions exemption program” that went into effect on 20th September 2021 [12].

Throughout this period of rising case counts and policy changes, Reddit users were reacting to and discussing events in real-time. Reddit is a social media platform where users can create posts with text, images, and videos on pages dedicated to a certain location or topic, called subreddits, that other users on the subreddit can then comment on or vote up or down [13]. For example, this study examined posts from the “Alberta: Wild Rose Country” subreddit (r/alberta), which at the time of writing has 181K active users [14]. Beyond an email address, no personal information is required to create an account, and as such, users can choose to be essentially anonymous [13]. Due to this anonymity, there is no concrete way of identifying demographics and characteristics of Reddit users outside of self-reporting. Additionally, some subreddits, such as r/The_Donald [15], allow for the assumption of certain characteristics about its users. In r/The_Donald, a subreddit that has now been banned for violating Reddit guidelines, it could be assumed that users were American individuals who support Donald Trump [15]. In the subreddit r/Alberta, it is possible to assume that users are Albertans. However, as subreddits are open to participation by any user, this cannot be confirmed. Further, some users on r/Alberta identify themselves as being from other provinces in their comments, though it is not possible to confirm the location of these individuals.

In recent years, Reddit has been increasingly viewed as a valuable source of information for monitoring health behaviors and public perceptions [16–21]. However, it is still considered a relatively new data source for social sciences research and has been used less frequently in research than more established social media platforms such as Twitter [13]. Furthermore, opinions towards “vaccine passports” as a form of vaccination enforcement remains a relatively unexplored research area.

The goal of this study is to understand the online opinions and beliefs towards mandatory vaccination policies in Alberta, Canada, between 1st September and 30th September 2021, by analyzing Reddit posts and comments from the r/alberta subreddit. This time period was chosen to ensure we captured opinions from before and immediately after the implementation of mandatory vaccines. Analysing opinions of mandatory vaccines and vaccination policies offers valuable insight into public perception that can be used to understand the themes underlying these discussions. The understanding of those themes could inform potential considerations by public health authorities surrounding mandatory vaccination policies.

## Methods

Data were collected from the subreddit r/Alberta. English Reddit posts were retrieved using Pushshift.io. Search terms were used to narrow results to only reflect conversations surrounding COVID-19 vaccines. These terms included “COVID-19,” “COVID,” “vaccine,” “vaccination,” “vax,” “jab,” “mandate,” and “passport.” Keywords were identified in consultation with the department librarian and with experts in novel social media infoveillance, authors HC. and ZB. Some other colloquial keywords were considered, including “viddy” and “corona,” but these terms did not yield any unique results and were thus excluded. Posts were included if they were created during the month of September 2021. This period was chosen to reflect the opinions relating to mandatory vaccines in Alberta both prior to their initial implementation and after their implementation.

**FIGURE 1:**
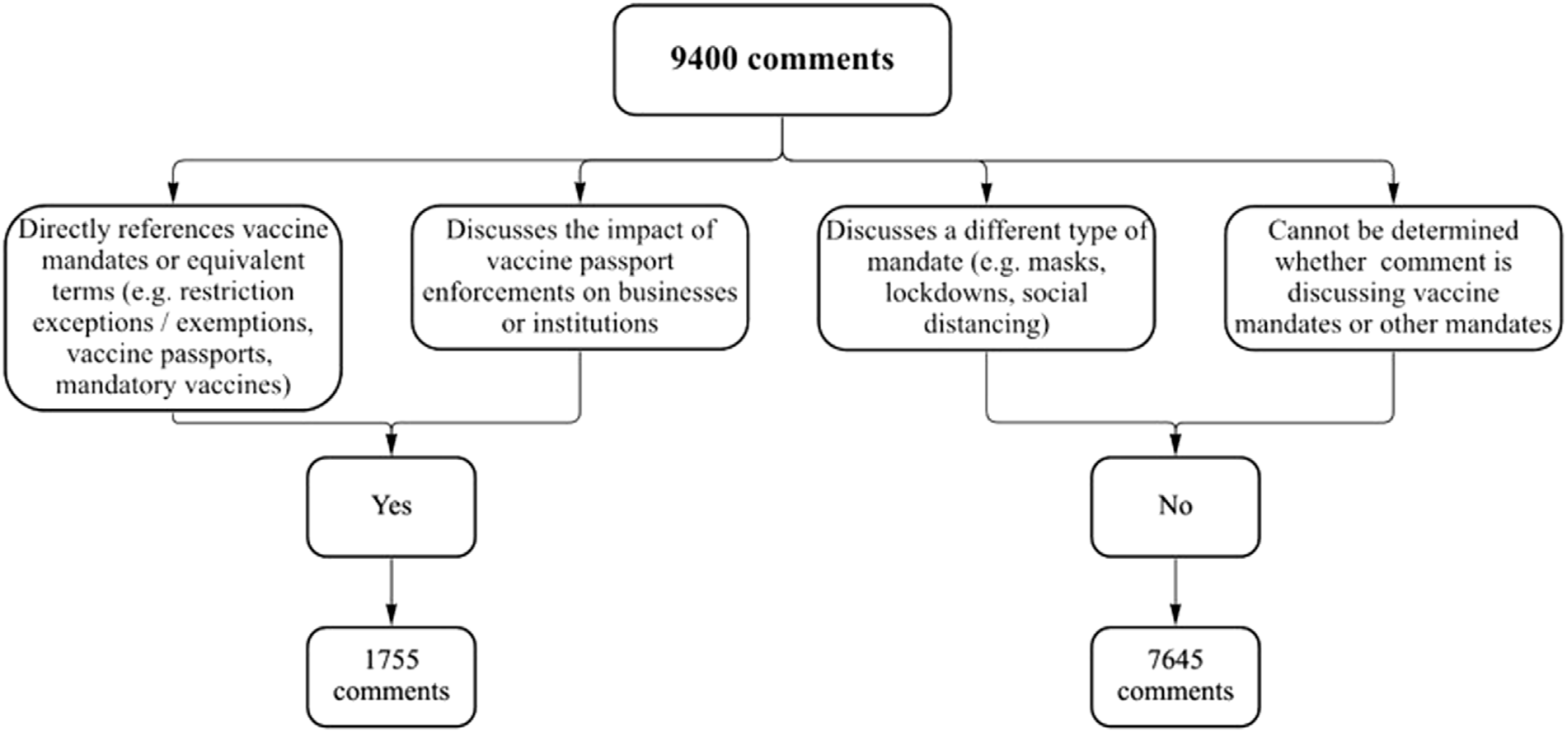
Screening criteria (Canada, 2021).

After the 9,400 comments were collected and organized into an Excel file, it was manually screened by three members of the research team. Screening was conducted to conclude if the comment specifically referred to vaccine mandates. In order to complete this screening, a list of conditions was developed to determine what comments would be included in the research. These conditions can be found in Figure 1. Out of those 9,400 comments collected, 1,755 were screened “yes” and were thus included in the study (i.e., included discussion about vaccine mandates). Once comments were screened, comments which included discussion of vaccine mandates or passports were copied into a separate excel file. Comments were then split in half randomly between BR and GBN. We followed Braun and Clarke’s approach to iterative qualitative analysis [22]. This method emphasizes the inclusion of any findings relevant to the research aim, regardless of the frequency of the code within the data. BR proceeded to code a random sample of 300 comments in order to develop a codebook using inductive coding methods outlined by Braun and Clarke, which was then discussed with and reviewed by other members of the research team [22]. BR and GBN then coded the entire dataset using said codebook. Finally, BR completed analysis by developing a list of five key themes with consultations from other researchers. Discrepancies were resolved by discussion between BR and GBN. The approach for data collection and analysis was guided by previously published work relating to online opinions [23–25].

In consultation with the University of Waterloo Research Ethics Board, it was determined that ethics approval was not needed for this research due to the publicly available nature of the data. In addition, quotes were paraphrased to maintain anonymity.

## Results

A summary of the five identified themes along with an example quote can be found in Table 1.

**TABLE 1:** Summary of key themes and example quotes (Canada, 2021).

Theme	Example quote
Sentiments Relating to Autonomy and Consent	Movement freedom. The passport is worse than a licence but not as bad as the star of David. It would be like prohibiting voting until everyone does an action they perceive as potentially damaging
Concerns About Passport Enforcement	Who enforces vaccination passports is one issue. Many retail personnel have been mistreated while being forced to enforce mask regulations. Even worse abuse might result from a vaccine passport, which effectively holds a piece of private medical information
Concerns About Government	Oh we’ll be getting passports for vaccinations. Also required masking. And business constraints. And because of Jason Kenney, Alberta will be the last province in Canada to receive them
Concerns About the Logistics of Vaccine Passports	I’m cool with passports, but using a third-party app, it will be difficult to convince me considering what happened with the Alberta Covid contact tracing app
Sentiments Relating to the Necessity of Passports to Avoid Lockdowns	Either a vaccine passport and increased vaccination rates, or it may actually be necessary to impose business closures and crowd size restrictions. Although it’s unfair to the vast majority of to who acted morally, it’s the truth

### Opinions Relating to Autonomy and Consent

Commenters described concerns relating to autonomy and consent, a opinion that stood regardless of their stance regarding mandatory vaccinations. Many commenters placed importance on bodily autonomy, explaining that mandatory vaccination campaigns would infringe on an individual’s right to choose to receive a vaccination instead of being mandated to receive one by the provincial government. These commenters typically equated vaccine passports with discrimination of the unvaccinated.

Movement freedom. The passport is worse than a licence but not as bad as the star of David. It would be like prohibiting voting until everyone does an action they perceive as potentially damaging.

Commenters expressed feeling that the lack of a vaccine mandate was infringing on the freedoms of vaccinated individuals, believing that it would be possible for restrictions to be lifted with the addition of a vaccine passport system. They described feeling as though they were being punished for following recommendations, while those who were unvaccinated prevented the majority of society from moving forward from the pandemic.

Yes, those who have received a vaccination want to carry on. Everybody understands how regulations hurt business and rising covid cases hurt healthcare infrastructure. A passport is protective of both.

I’m very angry right now. We (the vaccinated, which now comprises around 80% of the population) will suffer the consequences for the 20% of those who are too self-centered to receive vaccinations.

Commenters with opposing beliefs remained steadfast in their views that a passport would force “free individuals” to take a vaccine in order to participate in society.

Supporters of vaccine passports genuinely want to enact laws that are as burdensome as they can be on other people in an effort to force them to do something that they have freely chosen not to do.

Some expressed fears that Alberta is different from other provinces where a vaccine passport might be effective, believing that Albertans who had by then not received a vaccine would not concede to being immunized now. These fears likely stemmed from the proposed passport in Alberta having many exemption clauses, such as not requiring people to be vaccinated for a certain amount of time if they had recently contracted COVID-19.

Instead of half-assing it at the last minute and leaving businesses scrambling, we might have reopened gradually and carefully, maintained some precautions like masks, and had the vaccine passport ready to go. Almost every province has been able to reopen without our results.

### Concerns About Passport Enforcement

Commenters expressed concerns relating to potential harms to businesses if they were required to perform enforcement activities. Many stated that placing the burden of enforcement on individual businesses was purposefully done by the government to avoid anti-vaccine opinion being directed towards themselves. Moreover, the potential harms that minimum wage employees might experience due to needing to enforce vaccine passports was also a point of worry for many commenters. They felt it would not be fair to expose these individuals to abuse for doing their jobs.

They are transferring decision-making responsibility to individual companies. If you chose to enforce the passport, any of the customers who disagree with it will hold *you*, not the government, ultimately accountable. If they simply made it a law…

Who enforces vaccination passports is one issue. Many retail personnel have been mistreated while being forced to enforce mask regulations. Even worse abuse might result from a vaccine passport, which effectively holds a piece of private medical information.

Additionally, commenters stated that businesses might choose not to enforce vaccine passports in order to protect themselves against potential protests or loss of customers. It was suggested that this might not lead to a meaningful decrease in case counts, as customers who are unvaccinated would only frequent businesses that are not enforcing the passport. Commenters presented the idea that businesses were protecting themselves from further losses and harm, evident by the claims of businesses no longer enforcing masks.

Businesses will be reluctant to implement vaccine mandates if we leave it to them, either because they are themselves antivaxxers, or because they are frightened of losing more customers or having to deal with public outcry. If just some establishments demand it, the mounting cases won’t be addressed since unvaccinated customers will simply visit a competitor that will permit them admission, and the spread will continue. Additionally, it implies that companies that already need vaccinations will suffer as a result of their good intentions.

Because of this, many businesses and employees unofficially stopped enforcing mask laws. With the vaccine passport idea, we would run the risk of repeating it. To once again put that burden on those workers is just not fair.

Lastly, commenters described fears that a passport could not be enforced in a court of law. Citing previous cases where tickets for masking violations were dismissed in court, commenters expressed worry that similar things would happen with passport violations, leading to passports not being taken seriously or achieving their intent.

Another significant problem is going to be enforcement. We’ll watch what occurs with the enforcement of vaccine passports in British Columbia, Ontario, and Quebec. My proof of the absence of enforcement? Just take a look at Alberta in 2017! The few fines we did issue for disguising infractions or other rules violations were largely overturned in court. With police, it cannot be enforced. Any charges that people brought in court were not even attempted to be enforced or prosecuted by Alberta’s justice system. And trust me, those opposed to vaccinations and passports will litigate this in court.

### Concerns About Government

Commenters widely expressed frustration with the Alberta government surrounding their implementation of COVID-19 restrictions in general, as well as the late implementation of COVID-19 vaccine mandates. This frustration likely stems from the fact that commenters felt passports were necessary to curb the spread of COVID-19.

Oh we’ll be getting passports for vaccinations. Also required masking. And business constraints. And because of Jason Kenney, Alberta will be the last province in Canada to receive them.

Commenters also spoke about the vaccine passport not being referred to as a “vaccine passport.” Rather, the government made efforts to avoid referring to it as such, which some commenters saw as a transparent ploy to avoid the ire of their base.

When they made the announcement last week, the UCP was clear that this was not a vaccine passport. They literally dubbed it the “conveniently sized paper card.”

Additionally, commenters felt the government does not support the implementation of a vaccine passport, Jason Kenney publicly stated that he did not believe in the passport system.

Given that he isn’t requiring the vaccine or implementing a vaccine passport system, both of which would undoubtedly increase our immunisation rates… He is undoubtedly partially to blame.

These opinions were shared by commenters who felt that the Albertan government was corrupt. They accused the government of choosing to only implement vaccine measures cautiously so as to not jeopardize their political interests.

The introduction of vaccine passports would significantly shift the base of the UCP’s support to the Wildrose Independence Party. I think Hinshaw was making a reference to this at the end of the stream when he said it would be political suicide for Kenney. Clown world.

Lastly, some commenters conveyed opinions relating to government overreach. These commenters typically were anti-mandate, expressing that the implementation of a vaccine mandate was morally wrong and amounted to segregation.

They are defending segregation and a government mandate, which are both morally repugnant.

### Concerns About the Logistics of Vaccine Passports

Although less commonly discussed than other key themes, some commenters communicated fears that serious issues would follow the implementation of a vaccine passport, likely due to logistical concerns. Many spoke about issues with the potential technology that might be required to have a vaccine passport system not functioning properly. These fears stemmed from issues with other technologies put in place to combat the spread, such as the various COVID-19 contact tracing smartphone applications available in Canada.

I’m cool with passports, but using a third-party app, it will be difficult to convince me considering what happened with the Alberta Covid contact tracing app.

Some commenters explained that they felt there would be too many loopholes in the proposed passport system, with some specifically addressing potential passport forging.

Although Kenney is adamantly opposed to anything being referred to as a “Vaccine Passport,” he did declare that people will be able to download and print a form that certifies their immunisation status. They’re going to give out a vaccine passport, but they won’t call it that especially since I know it can be easily faked.

There were also concerns expressed relating to equity at large. Commenters described their concerns that these policies would disproportionately hurt individuals who might not have access to the technology to keep and display a vaccine passport. Specifically, commenters stated that issues could arise for individuals without access to a smartphone.

However, since it can be updated instantly and is inexpensive to deploy, an app will probably be the form of vaccine passport. For others who would not have access to a smartphone, such as some of the impoverished, the homeless, or the elderly, this could be a hurdle. If a passport system were to be implemented, there should be a backup plan in place in case the primary passport form proves to be too challenging. It should also be noted that vaccination status should always be communicated, regardless of the format used.

### Opinions Relating to the Necessity of Passports to Avoid Lockdowns

Commenters expressed immense frustration regarding the lack of vaccine passports forcing vaccinated individuals to be limited in their non-essential activities.

Either a vaccine passport and increased vaccination rates, or it may actually be necessary to impose business closures and crowd size restrictions. Although it’s unfair to the vast majority of us who acted morally, it’s the truth.

Some commenters disagreed, stating private businesses should have the option to implement a vaccine requirement if they choose, but it should not be widely mandated by the government.

Allowing only vaccinated people inside their establishments is a choice that private businesses are free to make. Make sure that their policy is clearly publicised so that we can decide whether or not to enter.

Many individuals went further than supporting a passport for non-essential businesses, such as retail shops, cosmetic spas or restaurants, suggesting potential healthcare consequences for individuals who are unvaccinated.

The next step up from vaccine passports is healthcare premiums for the unvaccinated, which I would support.

Paid in advance by a deadline, and only returned after receiving the whole vaccine course. If not, the money can be combined to increase the resources available to our underfunded healthcare system.

Some commenters went even further, expressing belief that unvaccinated individuals who get sick should not be treated in the hospital.

A hospital vaccine passport ought to exist. Let the antivaxxers contract the disease; society will be better off without them. Just natural selection.

## Discussion

The data suggests generally favorable opinions towards the potential of a vaccine passport in Alberta, a measure which was ultimately implemented on 15 September, 2021 [26]. The dialogue on Reddit mainly supported the implementation of a passport, though many individuals spoke about concerns relating to its implementation and logistics. Our data is consistent with the limited available literature in that most opinions towards vaccine mandates were positive [27–29].

Our findings are aligned with existing literature in that negative opinion relating to vaccine passports is often related to issues with bodily autonomy and freedoms [28, 30]. These individuals, though vastly outnumbered by passport supporters, claimed that a passport would inherently discriminate against the unvaccinated. However, our data did dissent from the literature in that anti-passport opinion was typically more reserved compared to some of the pro-passport opinion [28]. For example, while anti-passport comments generally argued about their right to choose what to put in their bodies, some pro-passport individuals went as far to suggest unvaccinated individuals should not have access to healthcare. Anti-passport opinion found in the literature focused on large conspiracy theories and intense anti-vaccine beliefs, specifically in terms of privacy and surveillance [28, 30]. This dissent could be due to moderator removal of more extreme anti-passport opinion present due to the culture of Reddit. Reddit is sometimes perceived by users as being a platform where posters and commenters are more comfortable sharing controversial and potentially harmful opinions due to the protection of anonymity [31]. Therefore, moderators of subreddits who support vaccine mandates might remove extreme content.

The data also suggests reservations relating to the actual enforcement of the passport, as well as concerns relating to both the government implementation of the passport and the logistical issues of the passport system. There is currently a lack of published literature surrounding these concerns, as most focus on the potential ethical and human rights concerns of a vaccine passport system. Novel insights from this data included understanding concerns held by individuals who supported the idea of a passport, specifically surrounding implementation and logistics.

In terms of enforcement, many commenters described feeling as though the onus of enforcing the passport was being put on businesses alone rather than the provincial government. This could suggest a general unhappiness with the provincial government in Alberta, which was confirmed in comments which directly spoke against the government. Commenters expressed frustration and anger that the premier did not support a potential vaccine passport and the general rollout of COVID-19 preventative measures. Commenters also stated that the Albertan government must be corrupt, suggesting a deep distrust of both the premier himself, as well as the government more broadly. This finding is very consistent with other COVID-19 vaccine hesitancy literature in general, where distrust in government is a common theme [32–35]. However, novel to this data is the distrust in government being displayed by both anti-vaccine individuals and pro-vaccine individuals. As stated previously, this could be due to the province’s mishandling of the pandemic, having almost triple the case counts of all other provinces (excluding Saskatchewan) [8–11]. The data also spoke to issues with logistics of a vaccine passport, mostly surrounding potential issues with technology required to access the passport. However, these issues were partially mitigated through the implementation of paper vaccine certificates, though there were still individuals who could potentially struggle with applying for a card online.

Our findings indicate support for vaccine passports in this sample of Reddit users, with most commenters stating they support vaccine mandates and requesting their implementation in order to resume pre-pandemic activities. Many were extremely supportive of passports, including implementing stricter measures than those put into place, including suggesting the use of passports for healthcare. This could be explained by the general frustration many individuals expressed surrounding their inability to participate in non-essential activities even after following all public health measures and waiting for the release of a vaccine. Additionally, these users might have felt frustration with their provincial government. These commenters also described a large frustration with unvaccinated individuals, claiming they were at fault for the inability to move out of the pandemic public health measures.

Limitations of this study include issues related to removed content. While Reddit itself does not have a policy in place against misinformation, community moderators, including those from r/Alberta, can choose to remove anti-vaccine misinformation from their subreddits. When a moderator removes a comment, it is no longer visible, thus there is no way to identify why said comment was removed. Therefore, even though our data presented as overwhelmingly positive opinions towards vaccine passports, it is possible that comments that did not support the passport were removed. Additionally, as this study is qualitative in methodology, it is not generalizable to the population of Alberta. This is also true as Reddit is anonymous, so user characteristics cannot be collected and analyzed, nor can it be guaranteed that all commenters are in actuality residents of Alberta. Finally, data were from a 1 month period, where the vaccine passport system was implemented in Alberta halfway through the month [12], This might have affected results either positively or negatively, with users not yet experiencing the actual implementation of the passport system. While this was captured in the second half of the month, there were also simply fewer comments discussing the passport once it had been implemented.

To our knowledge, this study is the first to discuss vaccine passport beliefs in Alberta using social media. This work can be used to inform potential considerations surrounding public perception of mandatory vaccines. Public health authorities must share clear, concise messaging about potential passport systems before they are implemented and develop reliable enforcement strategies. The results of this study can be utilized to further explore whether there is a correlation between public opinion and the effectiveness of stringent public health interventions, such as vaccine passports, during a health crisis. Furthermore, future research could consider data from multiple provinces at the time of vaccine passport implementation to determine if similar opinions are shared across provincial subreddits.
